# Baseline characteristics and recruitment for SWOG S1820: altering intake, managing bowel symptoms in survivors of rectal cancer (AIMS-RC)

**DOI:** 10.1007/s00520-024-08527-x

**Published:** 2024-05-22

**Authors:** Virginia Sun, Cynthia A. Thomson, Tracy E. Crane, Kathryn B. Arnold, Katherine A. Guthrie, Sarah G. Freylersythe, Christa Braun-Inglis, Lee Jones, Joseph C. Carmichael, Craig Messick, Devin Flaherty, Samir Ambrale, Stacey A. Cohen, Robert S. Krouse

**Affiliations:** 1 Department of Population Sciences and Department of Surgery, City of Hope, Duarte, CA 91010 USA; 2 University of Arizona Cancer Center, Tucson, AZ USA; 3 Division of Medical Oncology, Miller School of Medicine, University of Miami Sylvester Comprehensive Cancer Center, Miami, FL USA; 4 SWOG Statistics and Data Management Center, Fred Hutchinson Cancer Center, Seattle, WA USA; 5 University of Hawaii Cancer Center, Honolulu, HI USA; 6 Research Advocate, Arlington, VA USA; 7 Division of Colon & Rectal Surgery, Department of Surgery, University of California Irvine, Irvine, CA USA; 8 Department of Colon and Rectum Surgery, Division of Surgery, The University of Texas MD Anderson Cancer Center, Houston, TX USA; 9 ValleyHealth/Winchester Medical Center, Winchester, VA USA; 10 FHP Health Center, Guam, USA; 11 University of Washington and Fred Hutchinson Cancer Center, Seattle, WA USA; 12 Perelman School of Medicine, University of Pennsylvania and the Corporal Michael J. Crescenz Veterans Affairs Medical Center, Philadelphia, PA USA

**Keywords:** Quality of life, Bowel dysfunction, Intervention, Self-management, Diet modification

## Abstract

**Purpose:**

Many survivors of rectal cancer experience persistent bowel dysfunction. There are few evidence-based symptom management interventions to improve bowel control. The purpose of this study is to describe recruitment and pre-randomization baseline sociodemographic, health status, and clinical characteristics for SWOG S1820, a trial of the Altering Intake, Managing Symptoms in Rectal Cancer (AIMS-RC) intervention.

**Methods:**

SWOG S1820 aimed to determine the preliminary efficacy, feasibility, and acceptability of AIMS-RC, a symptom management intervention for bowel health, comparing intervention to attention control. Survivors with a history of cancers of the rectosigmoid colon or rectum, within 6–24 months of primary treatment completion, with a post-surgical permanent ostomy or anastomosis, and over 18 years of age were enrolled. Outcomes included total bowel function, low anterior resection syndrome, quality of life, motivation for managing bowel health, self-efficacy for managing symptoms, positive and negative affect, and study feasibility and acceptability.

**Results:**

The trial completed accrual over a 29-month period and enrolled 117 participants from 34 institutions across 17 states and one US Pacific territory. At baseline, most enrolled participants reported self-imposed diet adjustments after surgery, persistent dietary intolerances, and bowel discomfort post-treatment, with high levels of constipation and diarrhea (grades 1–4).

**Conclusions:**

SWOG S1820 was able to recruit, in a timely manner, a study cohort that is demographically representative of US survivors of rectal cancer. Baseline characteristics illustrate the connection between diet/eating and bowel symptoms post-treatment, with many participants reporting diet adjustments and persistent inability to be comfortable with dietary intake.

**ClinicalTrials.gov registration date:**

12/19/2019.

**ClinicalTrials.gov Identifier:**

NCT#04205955.

**Supplementary Information:**

The online version contains supplementary material available at 10.1007/s00520-024-08527-x.

## Introduction

In the USA, there are over 1.4 million people living with a history of colorectal cancer, with a rapid shift of rising incidence in adults younger than 50 years of age [1]. For rectal cancer, the current 5-year survival rate is 67%, reflecting advances in treatment and early detection [2]. Standard multimodal treatment for rectal cancer involves the sequenced combination of chemotherapy, radiotherapy, and surgery. Depending on tumor characteristics, surgical treatments often involve rectal anastomosis, creation of a permanent ostomy, or a temporary protective diverting ileostomy or colostomy that may be reversed in a second procedure.

Survivors of rectal cancer often experience persistent long-term effects of treatments that impact the quality of their survivorship. A common and debilitating long-term effect of rectal cancer treatment is bowel dysfunction. The constellation of postoperative bowel symptoms is known as low anterior resection syndrome (LARS), which includes fecal incontinence, frequency, urgency, a sense of incomplete fecal evacuation, and flatulence [3–6]. Symptom characteristics are dynamic, with wide variation in frequency and severity. In long-term survivors, prevalence rates of bowel dysfunction range from 27 to 56% [3, 5, 7–9], and symptom management is often challenging with few treatment strategies that are evidence-based [10]. Efforts to identify effective symptom management strategies are needed to enhance the quality of survivorship [11].

In the absence of high-level evidence-based treatments, survivors of rectal cancer often use self-management strategies such as diet modification to achieve bowel control [12–15]. Dietary adjustments are often undertaken without structured guidance, and survivors often avoid high quality, nutrient dense, cancer preventative foods such as vegetables, fruits, and whole grains, due to perceived risks of associated bowel problems with these foods [14]. This has the potential to increase risks of cancer recurrence and other comorbid health issues during the survivorship period.

SWOG S1820 was designed to determine the preliminary efficacy, feasibility, and acceptability of Altering Intake, Managing Symptoms in Rectal Cancer (AIMS-RC), a diet modification intervention to attenuate and alleviate bowel dysfunction during post-treatment survivorship. The purpose of this paper is to describe data on recruitment and pre-randomization baseline sociodemographic and clinical characteristics of SWOG S1820 participants.

## Materials and methods

The trial protocol has been previously described [16]. SWOG S1820 was a multisite, randomized trial conducted through the National Community Oncology Research Program (NCORP) research base of the SWOG Cancer Research Network, a National Cancer Institute (NCI)-supported National Clinical Trial Network (NCTN).

Survivors were eligible for the study based on the following criteria: (1) prior history of cancers of the rectosigmoid colon or rectum; (2) within 6–24 months after primary treatment completion; (3) have a post-surgical permanent ostomy (by definition living with bowel dysfunction); (4) anastomosis with LARS score of 21–42 (minor to major symptoms) for survivors with anastomosis; (5) be able to read, write, and speak English; and (6) over 18 years of age. The trial included both survivors with ostomy and anastomosis to be inclusive of the entire population of rectal cancer survivors with bowel dysfunction. Survivors were not eligible for the study if they were currently undergoing treatment for another cancer or were diagnosed with inflammatory bowel disease (ulcerative colitis or Crohn’s disease). The exclusion criteria were selected to minimize overlapping symptoms from other treatments or conditions. The study was approved by the NCI’s Cancer Control and Prevention Central Institutional Review Board (CIRB), and all participants provided signed informed consents.

**Fig. 1:**
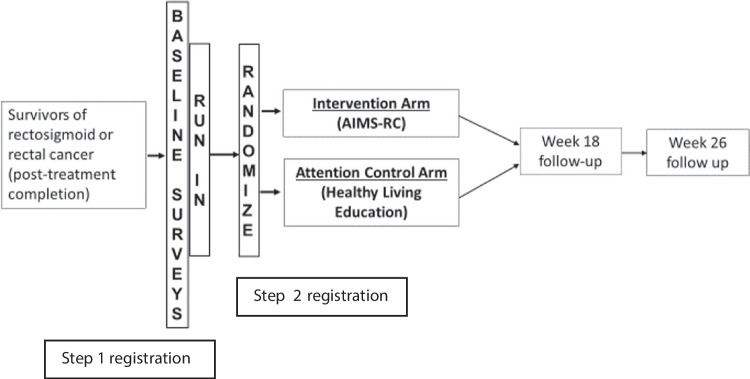
SWOG S1820 study schema

A two-step participant registration process was used (Fig. 1). After informed consent, step 1 registration occurred, and participants started the 14-to-21-day run-in period. The run-in and related activities were included to assess and enhance adherence to the study activities post-randomization. Run-in participants received a run-in packet from their clinic that included instructions, a 3-day food/symptom diary, and a postage-paid envelope. Participant information was securely shared with the University of Arizona, and the study coordinator reached out to complete an introductory study call within 48 h of receiving their information. The study coordinator instructed participants to complete the 3-day food/symptom diary and to return it by mail or email within 7 days of completion. The study coordinator also facilitated a telephone call from trained research assistants at the Behavior Measurement and Interventions Shared Resource (BMISR) of the University of Arizona Cancer Center to complete the Memorial Sloan-Kettering Bowel Function Instrument (MSK-BFI) questionnaire [17, 18] and a 24-h dietary recall (USDA multi-pass dietary recall methodology). Participants that successfully completed the introductory phone call, food/symptom diary, MSK-BFI, and 24-h dietary recall were registered to step 2 and randomized 1:1 to either the intervention or attention control arm, using a stratified (sex, ostomy status) and blocked randomization. For those that did not sufficiently complete the run-in activities, a resource manual with information on healthy living after cancer treatment was sent and there was no further study participation.

### Intervention and attention control designs

AIMS-RC is a telephone-based, social cognitive theory–driven intervention that is guided by the Motivation and Problem-Solving (MAPS) model of behavior change [19–22]. Each participant randomized to the intervention arm received ten telephone sessions centrally administered by trained health coaches from the University of Arizona over a 17-week period. In between the telephone sessions, intervention participants received smartphone text messaging (SMS) or email messaging to enhance engagement, promote behavior change, and support bowel symptom management goals. Each intervention participant also received a diet/symptom resource manual to support behavior change.

Participants randomized to the attention control arm received ten centrally administered telephone sessions over 17 weeks that focused on ten health promotion topics including national cancer survivorship guidelines on healthy living post-treatment (e.g., regular exercise, sun safety, hydration). The ten health promotion topics did not cover diet modification–related information. They also received SMS or email messaging with standard information on the ten healthy living topics and a healthy living resource manual. The information provided to the control arm did not address diet modification and bowel symptom management resources.

Several recruitment and retention strategies were implemented to promote enrollment site and participant engagement in the trial. The strategies were vetted with the SWOG Cancer Research Network Recruitment and Retention Committee and the core study team. All approaches were reviewed by a SWOG research advocate for feedback from the survivor’s perspective to ensure that all strategies were patient centered. Retention and engagement materials were used, including NCI Central IRB approved study brochures and quarterly newsletters specific to content by randomization assignment. To promote participating site engagement, monthly site coordinator calls were held throughout the study period to address site-specific queries on study process, problem-solving, and study engagement. Finally, study-specific pocket-sized protocol cards describing eligibility criteria were distributed for use by participating site investigators for screening of potential participants.

### Primary and secondary outcomes

The primary outcome for the trial was change in bowel function at 18 weeks post-randomization, as measured by the Memorial Sloan-Kettering Bowel Function Instrument (MSK-BFI) [17, 18] that is validated for use in both ostomy and anastomosis populations. Bowel function–related secondary outcomes included MSK-BFI total bowel function score at 26-week and MSK-BFI bowel function subscale scores (dietary, urgency, frequency) at 18 and 26 weeks. Other secondary outcomes were LARS score [23–27], quality of life (City of Hope-Quality of Life-Colorectal Cancer) [28], motivation (adapted Intrinsic and Extrinsic Motivation scale), self-efficacy (PROMIS Self-Efficacy for Managing Symptoms – Short Form 4a) [29], and positive/negative affect (I-PANAS-SF) [30, 31].

### Statistical analysis

A histogram was used to describe the distribution of participant enrollment over time. Baseline sociodemographic, clinical, and symptom characteristics were described for participants enrolled to step 1 registration (run-in). Medians with ranges and means with standard deviations were used to summarize continuous values; counts with percentages summarized categorical data.

## Results

**Fig. 2:**
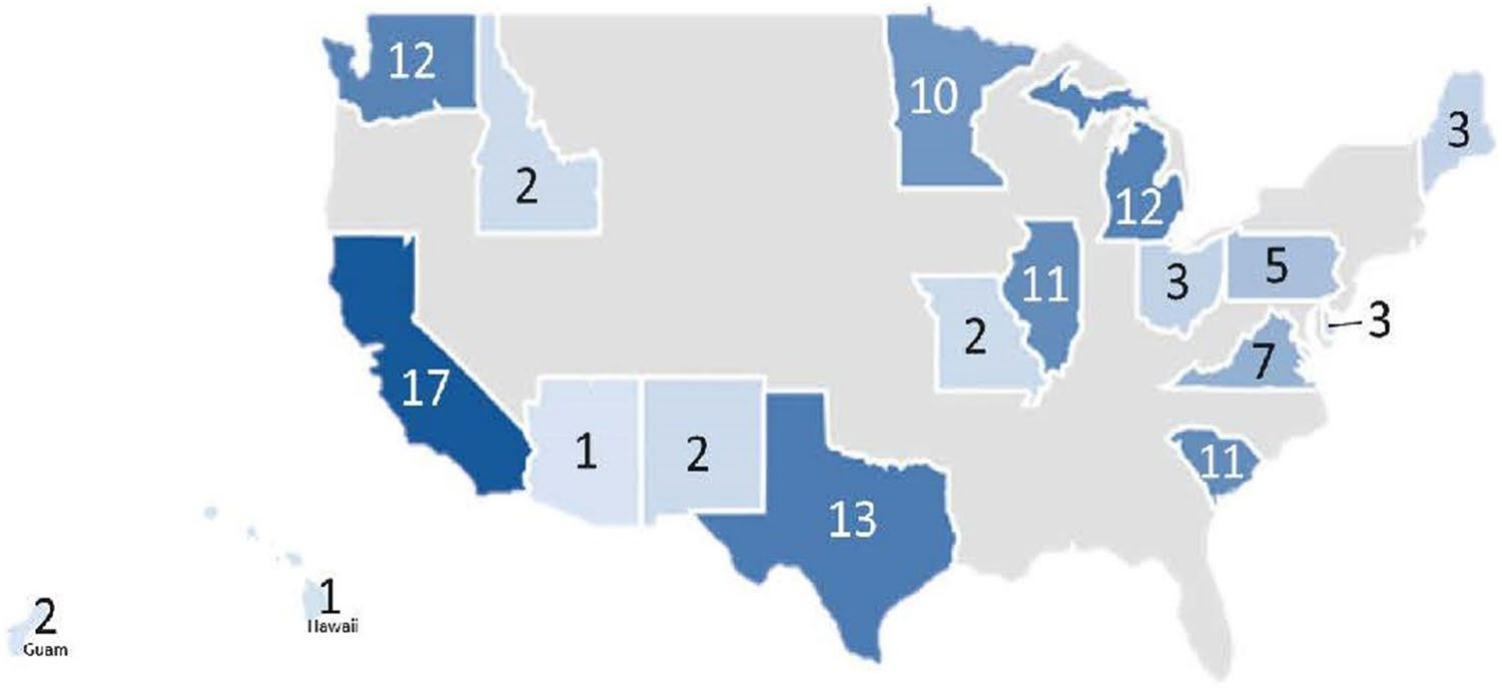
Distribution of SWOG S1820 participants by state

The trial enrolled and registered 117 participants to step 1 registration between February 2020 and April 2022 (23 months) (Fig. 2 Supplemental). With minor modifications, the trial continued with accrual nationally through the initial COVID-19 pandemic surge in 2020 and through subsequent surges. Participants from 17 states were included and the US territory of Guam, with the largest recruitment numbers from the states of California and Texas (Fig. 2). A total of 39 institutions contributed to overall enrollment, including 30 community institutions and nine academic institutions.

The median age at enrollment for the 117 consented participants was 55.2 years, with 54% female, 65% married or partnered, and 77% with at least some college education (Table 1). Most participants were overweight or obese (72%) and never smokers (68%). Most participants (75%) reported adjusting their diets following anastomosis/ostomy surgery, and 51% reported persistent discomfort with their diet after surgery without a clear plan for how to manage this.

**Table 1:** Baseline sociodemographic and health status characteristics of SWOG S1820 participants registered to step 1

Characteristic	All participants *N* = 117 *n* (%)	Ostomy participants *N* = 20 *n* (%)	Anastomosis participants *N* = 97 *n* (%)
Age (years)			
Median (range)	55.2 (26.6, 86.6)	58.0 (30.2, 78.1)	54.7 (26.6, 86.6)
Sex			
Female	63 (54)	9 (45)	54 (56)
Male	54 (46)	11 (55)	43 (44)
Race			
American Indian/Alaska Native	5 (4)	2 (10)	3 (3)
Asian	7 (6)	3 (15)	4 (4)
Black or African American	3 (3)	0 (0)	3 (3)
Native Hawaiian or other Pacific Islander	2 (2)	0 (0)	2 (2)
Unknown	3 (3)	0 (0)	3 (3)
White	97 (83)	15 (75)	82 (85)
Ethnicity			
Hispanic	9 (8)	2 (10)	7 (7)
Non-Hispanic	104 (89)	18 (90)	86 (89)
Unknown	4 (3)	0 (0)	4 (4)
Highest level of education			
Did not complete high school	2 (2)	0 (0)	2 (2)
Completed high school/GED/Vocational/secretarial/business	25 (21)	6 (30)	19 (20)
Any college	62 (53)	10 (50)	52 (54)
Any graduate school	28 (24)	4 (20)	24 (25)
Body mass index (BMI, kg/m^2^)			
Median (range)	27.5 (17.1, 66.3)	28.6 (17.1, 66.3)	27.3 (17.9, 52.3)
< 18.5 (underweight)	2 (2)	1 (5)	1 (1)
18.5– < 25 (normal weight)	30 (26)	3 (15)	27 (28)
25– < 30 (overweight)	41 (35)	8 (40)	33 (34)
≥ 30 (obese)	43 (37)	8 (40)	35 (36)
Missing	1	0	1
Smoking status			
Current	4 (3)	0 (0)	4 (4)
Former	28 (24)	7 (35)	21 (22)
Never	80 (68)	13 (65)	67 (69)
Unknown	5 (4)	0 (0)	5 (5)
Marital status			
Divorced	16 (14)	3 (15)	13 (13)
Married or partnered	76 (65)	13 (65)	63 (65)
Single	20 (17)	3 (15)	17 (18)
Widowed	5 (4)	1 (5)	4 (4)
Any change in marital status since diagnosis			
Yes	6 (5)	0 (0)	6 (6)
No	111 (95)	20 (100)	91 (94)
Adjusted diet because of surgery/ostomy			
Yes	88 (75)	16 (80)	72 (74)
No	28 (24)	4 (20)	24 (25)
Not answered	1 (1)	0 (0)	1 (1)
Time to comfort with diet after surgery/ostomy			
Less than 1 month	16 (14)	7 (35)	9 (9)
1 to 12 months	34 (29)	6 (30)	28 (29)
More than 12 months	6 (5)	1 (5)	5 (5)
I am still not comfortable	60 (51)	6 (30)	54 (56)
Not answered	1 (1)	0 (0)	1 (1)

Rectal cancer was the most common type of cancer (81%), followed by rectosigmoid colon cancer (17%) (Table 2). The majority of participants (68%) were diagnosed with stage III (T3) disease, and median time since surgery was 13.1 months. Ninety-seven participants (83%) had an anastomosis, and low anterior resection was the most common type of surgery (75%). At the time of enrollment, 40% of participants had grades 1–2 (mild to moderate) constipation, 53% had grades 1–2 (mild to moderate) diarrhea, and 13% had grades 3–4 (severe) diarrhea (Table 3).

**Table 2:** Baseline clinical and treatment characteristics for SWOG S1820 participants registered to step 1

Characteristic	All participants *N* = 117 *n* (%)	Ostomy participants *N* = 20 *n* (%)	Anastomosis participants *N* = 97 *n* (%)
Time since diagnosis (months)			
Median (range)	22.0 (7.1, 56.2)	24.1 (14.6, 38.3)	21.9 (7.1, 56.2)
Type of cancer			
Rectal	95 (81)	19 (95)	76 (78)
Rectosigmoid colon	20 (17)	1 (5)	19 (20)
Sigmoid colon	1 (1)	0 (0)	1 (1)
Other	1 (1)	0 (0)	1 (1)
AJCC clinical stage at diagnosis			
T stage			
T0	1 (1)	0 (0)	1 (1)
T1	9 (8)	1 (5)	8 (8)
T2	17 (15)	2 (10)	15 (15)
T3	79 (68)	15 (75)	64 (66)
T4	10 (9)	2 (10)	8 (8)
TX	1 (1)	0 (0)	1 (1)
N stage			
N0	44 (38)	7 (35)	37 (38)
N1	40 (34)	8 (40)	32 (33)
N2	27 (23)	4 (20)	23 (24)
Nx	6 (5)	1 (5)	5 (5)
M stage			
M0	107 (94)	17 (85)	90 (96)
M1	2 (2)	1 (5)	1 (1)
MX	5 (4)	2 (10)	3 (3)
Missing	3	0	3
Prior treatments			
Any chemotherapy	103 (88)	20 (100)	83 (86)
Any radiation therapy	85 (73)	19 (95)	66 (68)
Prior surgery related to this cancer			
Primary anastomosis	49 (42)	0 (0)	49 (51)
Ostomy	20 (17)	20 (100)	0 (0)
Temporary ostomy and re-anastomosis	48 (41)	0 (0)	48 (49)
Time since surgery (months)			
Median (range)	13.1 (4.4, 51.4)	13.3 (6.1, 32.0)	13.1 (4.4, 51.4)
Type of low anterior resection surgery			
Abdominoperineal resection	21 (18)	18 (90)	3 (3)
Low anterior resection	91 (78)	2 (10)	89 (92)
Sigmoid colectomy	5 (4)	0 (0)	5 (5)
LAR syndrome (LARS) burden[1]			
Minor LARS	15 (15)	0 (0)	15 (15)
Major LARS	82 (85)	0 (0)	82 (85)
Not applicable, ostomy	20	20	0 (0)
Zubrod performance status			
0	87 (77)	17 (85)	70 (75)
1	25 (22)	3 (15)	22 (24)
2	1 (1)	0 (0)	1 (1)
Missing	4	0	4
Current medications			
Antibiotics	2 (2)	0 (0)	2 (2)
Antidiarrheal medications	36 (31)	1 (5)	35 (36)
Medications for constipation	27 (23)	0 (0)	27 (28)
Probiotics	19 (16)	2 (10)	17 (18)
Used meditation, mindfulness, acupuncture, or other alternative therapies for bowel issues in past 5 months			
Yes	9 (8)	2 (11)	7 (7)
No	107 (92)	17 (89)	90 (93)
Missing	1	1	0

[1] LAR = lower anterior resection. LARS score. Valid for anastomosis patients only. Score range 0–42: no LARS (0–20), minor LARS (21–29), major LARS (30–42)

**Table 3:** Baseline gastrointestinal symptom severity for SWOG S1820 participants registered to step 1

	Percentage of evaluated patients by CTCAE grade								
	All participants[1] *N* = 117			Ostomy participants *N* = 20			Anastomosis participants[1] *N* = 97		
	Grade 0 [2]	Grades 1–2	Grades 3–4	Grade 0 [2]	Grades 1–2	Grades 3–4	Grades 0 [2]	Grades 1–2	Grades 3–4
Constipation	60	40	0	80	20	0	56	44	0
Dehydration	83	17	0	90	10	0	82	18	0
Diarrhea	34	53	13	65	35	0	28	56	16
Fecal incontinence	49	50	1	90	10	0	41	58	1
Flatulence	28	72	1	35	65	0	26	73	1
GI pain	66	34	0	80	20	0	63	37	0
Nausea	92	8	0	90	10	0	93	7	0
Vomiting	97	3	0	95	5	0	97	3	0

[1] One participant did not have a symptom assessment at baseline, and one participant did not have dehydration assessed. [2] Grade 0 = symptom not present

At enrollment among participants with anastomosis, the mean LARS score was 35.5 (Table 4). For bowel function scores, participants with an ostomy reported higher subscale and total scores, although the difference by ostomy status is small. Mean quality of life scores, including the four domains (physical, psychological, social, spiritual) varied between 5.2 and 7.1. Overall, negative affect (mean = 10.1) was low at enrollment (score range of 10–50, with lower scores representing lower levels of negative affect).

**Table 4:** Baseline outcome measure scores for SWOG S1820 participants registered to step 1

Outcome measure	All participants *N* = 117 Mean (SD)	Ostomy participants *N* = 20 Mean (SD)	Anastomosis participants *N* = 97 Mean (SD)
Self-efficacy for managing symptoms [1]	44.1 (7.8)	47.9 (8.2)	43.4 (7.5)
Bowel function [2]			
Dietary subscale	13.7 (3.3)	15.4 (3.3)	13.4 (3.2)
Frequency subscale	N/A	N/A	19.1 (3.7)
Urgency/soilage subscale	15.0 (3.8)	17.7 (3.1)	14.5 (3.7)
Global score	28.7 (5.9)	33.0 (5.4)	27.8 (5.6)
Missing	12	3	9
Quality of life [3]			
Physical	6.3 (1.9)	6.9 (1.6)	6.1 (1.9)
Psychological	6.8 (1.7)	7.0 (1.4)	6.7 (1.7)
Social adjustment to ostomy	5.4 (2.4)	6.3 (1.8)	5.2 (2.5)
General quality of spiritual well-being	7.1 (2.0)	6.8 (1.8)	7.1 (2.0)
Total	6.4 (1.6)	6.8 (1.3)	6.3 (1.6)
Motivation for managing bowel health [4]	32.7 (8.9)	32.3 (9.5)	32.8 (8.8)
Affect [5]			
Positive	18.4 (3.6)	18.9 (4.2)	18.2 (3.5)
Negative	10.1 (4.4)	8.9 (3.6)	10.4 (4.6)

[1] PROMIS = Patient-Reported Outcomes Measurement Information System. Score range 0–100. Higher scores indicate greater self-efficacy. [2] MSK-BFI = Memorial-Sloan Kettering Cancer Center Bowel Function Instrument. Score range 18–90. Higher scores indicate better bowel function. Frequency subscale is valid for anastomosis participants only. [3] COH-QOL-CRC = City of Hope Quality of Life—Colorectal Cancer. Score range 1–10. Higher scores indicate higher quality of life. [4] Score range 0–40. Higher scores indicate higher motivation. [5] I-PANAS-SF = International Positive And Negative Affect Short Form. Score range 5–25. Higher scores indicate greater affect

## Discussion

AIMS-RC is one of the first and few interventions to systematically address dietary behavior changes to improve bowel symptoms in rectal cancer. The recruitment data confirms the feasibility of conducting complex behavior change–driven symptom management trials through national cancer research networks such as NCI NCORP and SWOG, and the ability to complete enrollment in a timely manner. Representation from academic (*N* = 9) and community (*N* = 30) oncology practice settings potentially enhances generalizability of study results and allows for the enrollment of a representative population across broad regions of the country, stretching from the Northeast (Maine) to the US Pacific territories (Guam). The participant characteristics indicated that the sample was representative of survivors with the disease by age, based on recent trends showing a substantial increase of younger onset colorectal cancer incidence in the USA, particularly in adults ≤ 50 years [1, 32]. The relatively equal representation by sex among study participants provides an opportunity to explore the impact of sex on potential outcome differences in dietary behavior change interventions.

The baseline characteristics illustrate the connection between diet/eating and bowel symptoms after surgery and other treatments. The large proportion of participants that reported diet adjustments after surgery and persistent inability to be comfortable with dietary intake post-treatment suggest that dietary adjustments are common in survivors of rectal cancer regardless of ostomy status, an observation described previously by this research team [15]. Major LARS was common at enrollment for study participants, and higher levels of grades 1–4 constipation and diarrhea were observed. These baseline characteristics in a sample of longer term (6–24 months) survivors of rectal cancer underscores the importance of addressing post-treatment bowel symptom control. Additionally, a large proportion of participants were overweight (35%) and obese (37%). Current evidence suggests that survivors of cancer fall short in achieving national survivorship dietary guidelines [33].

Several aspects of the S1820 study design were included to promote recruitment and adherence and reduce loss to follow-up. The pre-randomization run-in period was designed to provide enrolled participants who were registered at step 1 with an overview of the key strategies used in AIMS-RC, including completion of the food and symptom diary and telephone communication with study health coaches. It was also designed to improve adherence and reduce attrition post-randomization. S1820 was able to successfully integrate a pre-randomization run-in period.

The centralized administration design of the AIMS-RC intervention was intended to promote high intervention fidelity and reduce participating site burden. Reductions in site burden contributed to S1820’s ability to continue with enrollment through multiple surges of the COVID-19 pandemic and complete accrual in a timely manner. Centralized intervention delivery allowed for more effective training of health coaches, with ease and efficiency in providing ongoing fidelity monitoring, coaching call documentation, and feedback. Telephone approaches offer many advantages, including increased access, affordability, and convenience for participants. The approach speaks to the potential for scalability of the AIMS-RC intervention to a larger population of survivors.

### Lessons learned

Throughout the study enrollment period, many opportunities were available to advance knowledge on the design and conduct of diet behavior change interventions through NCORP- and NCTN-funded networks like SWOG. Clarity in communicating workflows and responsibilities between participating site investigators, participating site staff, and the core research team and health coaches is essential to the successful conduct of complex behavior change interventions such as AIMS-RC. The workflows and responsibilities should be clearly outlined in study protocols. Participating site staff training is important to enhance communication on protocol workflows and responsibilities and should be designed in a virtual, asynchronous format (e.g., website) for easy access and completion. Early and regular engagement meetings with site investigators and site staff in the trial process are critical to providing forum to learn from participating sites and to identify protocol procedural challenges and solutions early. Timely responses to site staff questions about eligibility and workflows are important for site engagement and help with minimizing delays and deviations. Finally, regular meetings of the research team, with input from cancer patient advocates, helped to ensure that recruitment strategies were shared and any issues were addressed in a time sensitive manner.

## Conclusions

SWOG S1820 was able to complete national recruitment and deliver a centralized, telephone-based diet modification intervention for symptom management in post-treatment survivors of rectal cancer. The novel and unique design of the study afforded the opportunity to expand knowledge on designing and conducting national behavior change interventions.

### Supplementary Information

Below is the link to the electronic supplementary material.

Supplementary file1 (DOCX 129 KB)

## Data Availability

Data is available per NIH/NCI and SWOG Cancer Research Network policies and approval.
