# Supplementary figures and images for: Baseline characteristics and recruitment for SWOG S1820: altering intake, managing bowel symptoms in survivors of rectal cancer (AIMS-RC)
Source: Support Care Cancer. 2024 May 22;32(6):371. doi: 10.1007/s00520-024-08527-x (PMC11111552; doi:10.1007/s00520-024-08527-x)

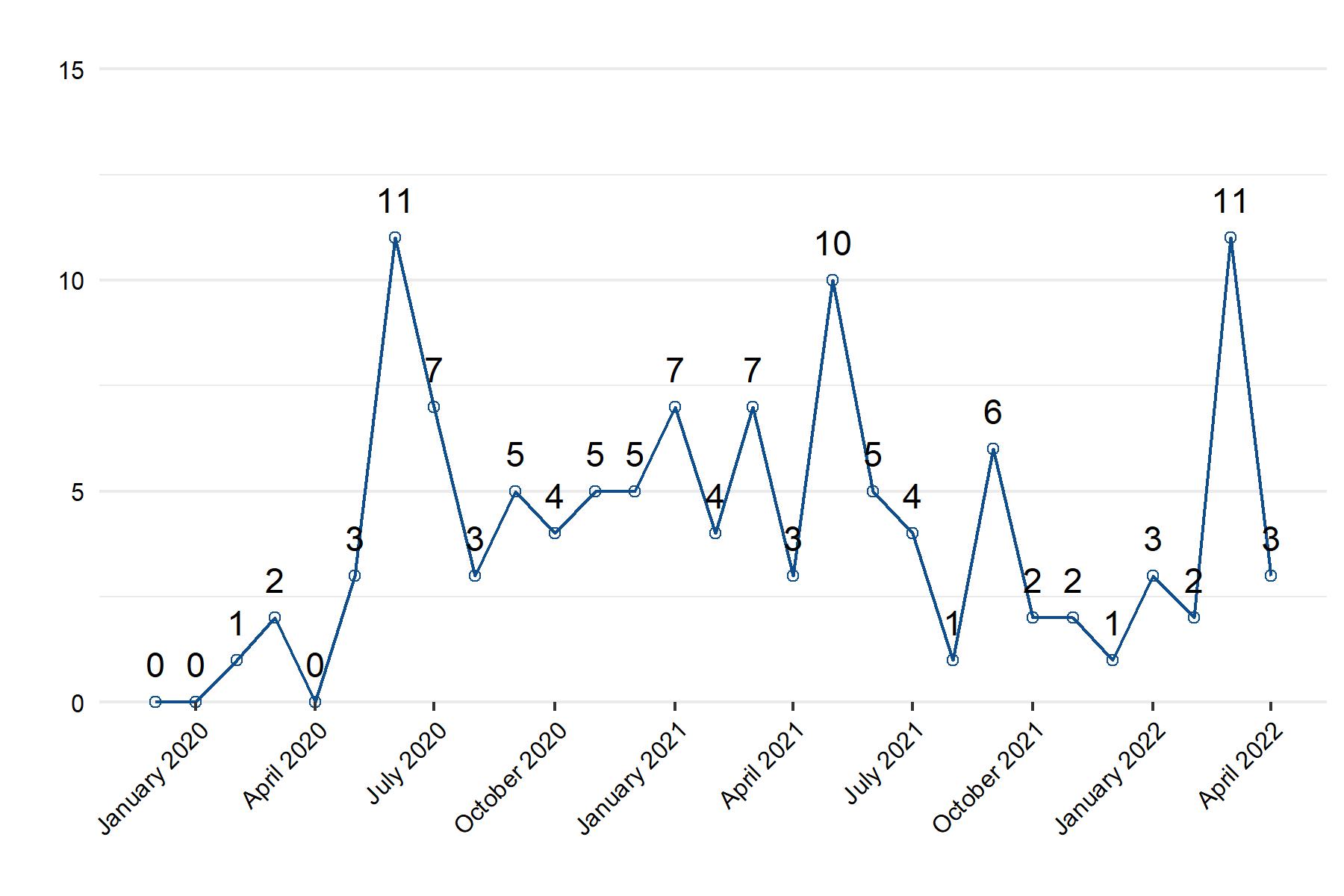


**Figure 2. S1820 Accrual to Run-In**

Supplement: Supplementary file 1 — Supplementary file1 (DOCX 129 KB) [file 520_2024_8527_MOESM1_ESM.docx]
